# The effect of three exercise approaches on health-related quality of life, and factors associated with its improvement in chronic whiplash-associated disorders: analysis of a randomized controlled trial

**DOI:** 10.1007/s11136-018-2004-3

**Published:** 2018-09-17

**Authors:** Maria Landén Ludvigsson, Gunnel Peterson, Anneli Peolsson

**Affiliations:** 1 Department of Medical and Health Sciences, Division of Physiotherapy, Linköping University, 58183 Linköping, Sweden; 2 Rehab Väst, Region Council of Östergötland, Department of Rehabilitation and Department of Medical and Health Sciences, Linköping University, Linköping, Sweden; 3 Centre for Clinical Research Sörmland, Uppsala University, Eskilstuna, Sweden

**Keywords:** Quality of life, Whiplash, Spine, Exercise, Physiotherapy, Chronic

## Abstract

**Purpose:**

The aim was to evaluate whether neck-specific exercise, with (NSEB) or without (NSE) a behavioural approach, improves health-related quality of life (HRQoL) compared to physical activity prescription (PPA) in chronic whiplash-associated disorders (WAD) grades 2 and 3. A secondary aim was to identify factors associated with HRQoL and HRQoL improvement following exercise interventions.

**Methods:**

This is a secondary analysis of a multicentre randomized clinical trial. Participants (*n* = 216) with chronic WAD grades 2 and 3 were randomized to 12 weeks of PPA or physiotherapist-led NSE or NSEB. The EQ-5D 3L/EQ-VAS and SF-36v2 physical (PCS) and mental (MCS) component summaries were collected together with several neck-related and psychosocial outcomes at baseline, after 3, 6 and 12 months, and were analysed with linear mixed models (all time points) and multivariate linear regressions (baseline, 6 months).

**Results:**

NSE/NSEB resulted in better outcomes than PPA (EQ-VAS and SF-36 PCS, both groups, *p* < 0.01) but not in a higher EQ-5D score. Improvement over time was seen in EQ-5D/EQ-VAS for the NSEB group (*p* < 0.01), and for NSE/NSEB as measured with the PCS (*p* < 0.01). Factors associated with baseline HRQoL and change to 6 months in HRQoL (*R*^2^ = 0.38–0.59) were both neck-related and psychosocial (e.g. depression, work ability).

**Conclusion:**

Neck-specific exercise, particularly with a behavioural approach, may have a more positive impact on HRQoL than physical activity prescription in chronic WAD grades 2 and 3. HRQoL is however complex, and other factors also need to be considered. Factors associated with HRQL and improvements in HRQoL following exercise are multidimensional.

Trial registration number: ClinicalTrials.gov, No. NCT01528579.

**Electronic supplementary material:**

The online version of this article (10.1007/s11136-018-2004-3) contains supplementary material, which is available to authorized users.

## Introduction

Neck pain is rated as the 6th leading global cause of years lived with disability, which is higher than for instance diabetes and ischemic heart disease [1]. One cause that presents a significant public health problem is whiplash-associated disorders (WAD). Hospital visits, impairment and disability due to WAD have increased, and the annual incidence of reported whiplash injuries is likely to be at least 300 per 100,000 [2–4]. The recovery rate after a whiplash injury in general is 50%, but among those with neurological deficits, 90% continue to report symptoms after 1 year [5–7]. People with chronic WAD report worse health than people with non-specific chronic neck pain [8, 9]. WAD thus has a clear impact on health-related quality of life (HRQoL). The worse the level of disability, the lower the HRQoL and the higher the costs for society regarding WAD [10]. Lower HRQoL in chronic WAD has also been associated with depression, pain catastrophizing and pain, as well as non-pain-related factors [11, 12]. Physical health in general is strongly associated with age, while mental health is reportedly less so [13]. Health is thus multidimensional, and in the general population comorbidities, age and low social class are the major factors suggested to impact HRQoL in several studies [14]. The World Health Organization defines health as “a state of complete physical, mental and social well-being and not merely absence of disease or infirmity” [15]. Chronic WAD involves a variety of symptoms with considerable overlap between physical and psychosocial origins, thus both aspects may need to be considered when choosing the appropriate treatment. In order to address psychological factors, cognitive behavioural components in physiotherapy management of chronic WAD have been suggested [16].

Looking further into the physical manifestations of longstanding WAD, dysfunction and characteristic fatty infiltration of predominantly the deep cervical muscles are reported [17–20]. Exercise of these muscles may thus be a feasible treatment. The positive impact general physical activity has on health is well established [21], but to our knowledge only one randomized controlled trial [22] of neck-specific exercise has included both chronic WAD grade 2 (with pain and local physical findings) and grade 3 (also including neurological findings) as defined by the Quebec Task Force [23]. The study reported reduced disability following neck-specific exercise without (= NSE) or with (= NSEB) a behavioural approach compared to physical activity prescription (PPA) [22, 24]. However, whether this also translates into better HRQoL in chronic WAD grades 2 and 3 has not previously been reported in the literature. Furthermore, to our knowledge factors that can explain improvement in health following exercise interventions in this group are also unknown.

The aim of this analysis was to evaluate whether neck-specific exercise, with or without a behavioural approach, improves HRQoL compared to physical activity prescription in chronic WAD grades 2 and 3. A secondary aim was to identify factors associated with HRQoL and HRQoL improvement, which can explain the variance of the improvement, following these three exercise interventions. We hypothesized that NSE/NSEB would have a more positive impact on HRQoL than PPA.

## Methods

### Design and procedure

**Fig. 1:**
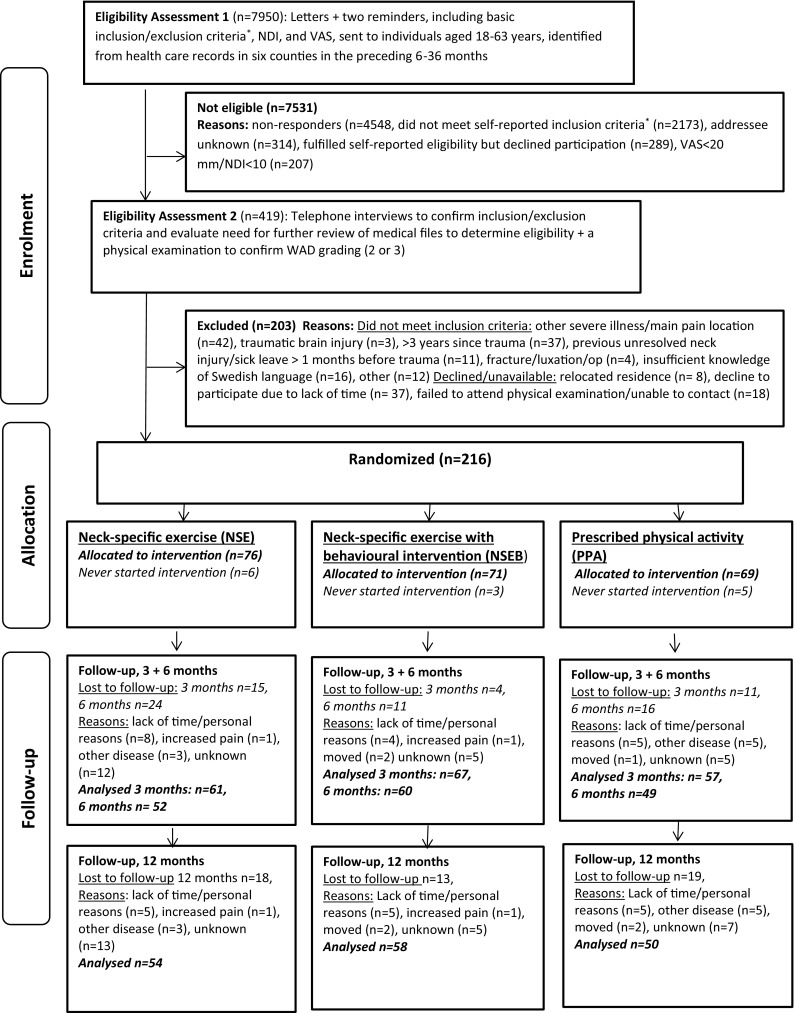
Flow chart of participants. The numbers indicate participants who answered the EQ-5D/SF-36 ^*^Whiplash injury in the preceding 6–36 months, reported to be the onset of current symptoms, unconsciousness/loss of memory in connection with the whiplash injury, previous neck trauma with unresolved symptoms, previous neck surgery, ongoing malignant disease, severe psychiatric disorders, drug abuse, difficulties understanding the Swedish language

This is a secondary analysis of a multicentre randomized clinical trial [22]. Informed consent was collected before randomization into one of three exercise groups, using a computer-generated list. It was handled by an independent researcher who put the results into opaque envelopes for further distribution to the treating physiotherapists. The data, collected at baseline and at 3, 6 and 12 months, were registered by another independent researcher. The number of participants at each time point is presented in Fig. 1.

### Participants

The 216 participants, aged 18–63, with WAD grade 2 or 3 for 6–36 months, were recruited in 2011–2012. Additional inclusion criteria were a Neck Disability Score (NDI) [25] of > 10/50 points and/or an average neck pain intensity over the past week on the Visual Analogue Scale (VAS) of > 20/100 mm. Exclusion criteria included previous neck trauma with unresolved symptoms, more dominant pain elsewhere, insufficient knowledge of the Swedish language and conditions that were potentially detrimental to completing the study interventions [22].

The mean age was 40.5 (range 18–63, SD 11.4) years, and 142 (65%) women and 74 (35%) men were included. Further baseline characteristics are presented in Table 1. Comorbidity was evaluated with question number 4 from the Work Ability Index (WAI) [26], where participants were asked to report whether they currently suffer from any other diseases (circulatory, respiratory, disease of the nervous system, eye/ear, gastrointestinal system, urinary system/genitals, skin, blood, tumours, metabolic disease, eating disorders, psychological distress, congenital disabling deformity or other).

**Table 1:** Baseline variables

Variable	NSE (*n* = 76)	NSEB (*n* = 71)	PPA (*n* = 69)	*p* value
WAD grade 2/3 [*n* (%)]	49/27 (64/36)	33/38 (46/54)	41/28 (58/42)	0.08
Gender female [*n* (%)]	57 (75)	47 (66)	38 (55)	0.04
Age, mean (range) SD	38 (18–62) 11.3	40 (19–63) 11.6	43 (19–63) 10.7	0.03
Months since injury, mean (range) SD	19 (6–36) 8.7	20 (6–36) 8.9	20 (6–36) 10.3	0.69
Smoker [*n* (%)]	17 (22)	8/62 (11/89)	12/55 (18/82)	0.22
Educational level [*n* (%)]				0.44
Educational level, elementary	4 (5)	6 (9)	6 (9)	
Educational level, high school	38 (50)	40 (57)	34 (51)	
Educational level, university	31 (41)	21 (30)	24 (36)	
Educational level, other	3 (4)	3 (4)	3 (4)	
Use of analgesic drugs yes [*n* (%)]	40 (53)	44 (62)	45 (67)	0.23
Employed [*n* (%)]	61 (80)	57 (80)	52 (75)	0.71
Physical activity level, IPAQ				0.74
Physical activity level, low	16 (30)	14 (26)	25 (34)	
Physical activity level, medium	26 (48)	25 (45)	26 (36)	
Physical activity level, high	12 (22)	16 (29)	22 (30)	
Comorbidity yes [*n* (%)]^a^	56 (74)	52 (73)	49 (71)	0.76
Mental disorder/distress	15 (20)	20 (29)	18 (27)	
Gastrointestinal disease	17 (23)	18 (27)	21 (32)	
Respiratory disease	17 (22)	12 (18)	6 (9)	
Skin disease	16 (21)	12 (18)	7 (11)	
Cardiovascular disease	8 (11)	8 (11)	6 (9)	
Endocrine/metabolic disease	9 (12)	8 (12)	11 (17)	
Neurological/sensory disease (eye, ear)	10 (13)	4 (6)	9 (13)	
Digestive disease	9 (12)	8 (12)	11 (17)	
Number of comorbidities, mean (SD)	1.8 (1.3)	1.7 (1.4)	1.8 (1.4)	0.90
Pain catastrophizing (PCSc), mean (SD)	19 (10)	20 (13)	17 (10)	0.46
HADS, depression, mean (SD)	4.5 (3.8)	4.8 (4.3)	5.0 (4.3)	0.89
HADS, anxiety, mean (SD)	5.8 (3.9)	5.9 (5.1)	6.3 (3.6)	0.78
Neck disability (NDI), mean (SD)	31 (12.5)	34 (13.6)	34 (13.7)	0.39
Current pain, VAS, mean (SD)	39 (24)	46 (24)	42 (26)	0.26
Pain disability (PDI), mean (SD)	19 (12)	23 (16)	20 (13)	0.30
Work ability (WAI), mean (SD)	35 (6)	36 (7)	35 (7)	0.64
Kinesophobia (TSK), mean (SD)	22 (5)	22 (6)	22 (6)	0.99

*HADS* Hospital Anxiety and Depression Scale, *IPAQ* International Physical Activity Questionnaire, *NDI* Neck Disability Index (% score), *NSE* neck-specific exercise, *NSEB* neck-specific exercise with behavioural approach, *PCSc* Pain Catastrophizing Scale, *PDI* Pain Disability Index, *PPA* prescription of physical activity, *SD* standard deviation, *TSK* Tampa Scale of Kinesiophobia, *VAS* Visual Analogue Scale, *WAD* whiplash-associated disorder

^a^Comorbidity: non-musculoskeletal diseases with > 5 participants in all of the groups are specified. Tumours, blood, genital/urinary disease, birth defects and “other” with < 5 participants and are not presented

### Interventions and settings

The interventions were led by experienced primary care physiotherapist in six Swedish counties. The physiotherapists were selected and matched to the interventions to work within their field of interest and knowledge as far as possible. A one-day training workshop was held by the project leaders. The workshops were matched to the interventions, and included manual end exercise training, theoretical information, and standardized oral and written information about the interventions. The timeframe and specific components of the interventions during the 12-week intervention period have been previously published [22], but are presented briefly below.

#### Neck-specific exercise (NSE)

Neck-specific exercise without pain provocation and with a focus on the deep cervical muscles was performed with a physiotherapist twice weekly, with additional home exercises. After initial unresisted activation of the deep muscles in lying and sitting, gym exercises without pain provocation were introduced, with progressive head resistance training using a weighted pulley, focusing on good posture and low load endurance. A detailed description of the exercises can be found at the Academic Archive On-line [27].

#### Neck-specific exercise with a behavioural approach (NSEB)

The exercises were the same as those undertaken by the NSE group, but in accordance with the concept of behavioural graded exercise, participants were encouraged not to focus on a temporary increase in neck pain, but rather on success in exercise progression [28]. They also received basic behavioural intervention training, led by the physiotherapist. This included oral education regarding physiological and psychological aspects of pain, as well as activities aimed at pain management (such as relaxation, breathing exercises, etc.), goal setting and problem-solving, including the management of symptomatic relapses. Patients were encouraged to consider what they learnt and practise relevant pain management skills, for instance relaxation exercises, at home between sessions [22].

#### Physical activity prescription (PPA)

Based on medical history and a short motivational interview [29], participants were prescribed individually tailored general physical activity (e.g. gym classes, Nordic walking) to be performed outside the health care system. One follow-up visit or phone call was encouraged.

### Outcomes

Two generic measurements were used to measure HRQoL, the EQ-5D 3L and the SF-36v2®. The two instruments do not provide interchangeable results for people with neck pain, unlike in many other clinical areas [30, 31]. Furthermore, the SF-36 can be presented as separate physical and mental health summaries, which is not the case with EQ-5D. Both measurements come with sets of preference weights obtained from the general population in the UK, using standard gamble (SF-36) and time-trade-off (EQ-5D) techniques. The EQ-5D index score can be used in cost-effectiveness analyses, while the SF-36 separated summary scores cannot.

The main outcome for this analysis was the EQ-5D questionnaire which is commonly used, and is recommended by the National Institute for Health and Clinical Excellence [NICE] [32] in cost-effectiveness analyses, which are becoming increasingly important in health care. The EQ-5D consists of a descriptive system and the EQ-VAS. The score is a five-dimensional health state classification [33] focusing on mobility, self-care, usual activities, pain/discomfort and anxiety/depression. Each dimension is assessed by one question on a three point scale (no problems, some problems, extreme problems) on the day the questionnaire is completed. The results are scored and converted to a single summary index between − 0.59 and 1.00, with 1.00 indicating ‘full health’ and 0 representing ‘dead’. Negative EQ-5D scores represent health states valued as worse than dead. The EQ-5D also consists of a separate vertical VAS scale (EQ-VAS) from 0 (worst imaginable health state) to 100 (best imaginable health state), which records the respondent’s self-rated health. Thus, other individual aspects of perceived health, not covered by the five questions, can also be considered. The EQ-5D is considered valid and reliable [34]. In general western populations, aged 18–64, the average EQ-5D scores are reported to range from 0.776 (older ages) to 0.979 (younger ages), and in EQ-VAS, 71 (older ages) to 89 (younger ages) [35]. Permission to use the EQ-5D was obtained from the EuroQol Group Foundation.

The secondary outcome was the valid and reliable Short Form 36 (SF-36v2) Health Survey [36, 37]. It contains 36 items, measuring eight health-related quality of life domains—physical functioning, role limitation (physical), bodily pain, general health, vitality, social functioning, role limitation (emotional) and mental health, with a four-week recall period. Scores from the eight domains are weighted and aggregated into two summary measures as t-scores, the physical component summary (PCS) and the mental component summary (MCS), where higher scores represent better health. In general western populations, mean PCS and MCS scores are reported to be around 51 [38], with a range from PCS 47/MCS 52 (ages 55–64) to PCS 54/MCS 46 (ages 18–24) [39]. A licence to use the SF-36 was obtained from Quality Metrics Inc., USA.

### Factors potentially associated with HRQoL and change in HRQoL score

To examine factors associated with baseline, and change in the EQ-5D index, EQ-VAS, PCS and MCS, scores from baseline and the six-month follow-up were chosen, where all self-reported measures of primary interest were collected. (At 3 months, the Hospital Anxiety and Depression Scale (HADS scored 0–21) [40] and the Tampa Scale for Kinesiophobia, TSK-11 short form, TSK scored 11–44 [41], were not collected). Factors were chosen from predictors presented in the literature of health in general, together with other measurements of relevance to longstanding WAD from our RCT study: gender, age, WAD grade, comorbidities (number), months since injury, neck pain intensity (VAS scale 0–100), neck-related disability (NDI, 0–50 [25]), pain catastrophizing (the Pain Catastrophizing Scale, (PCSc) 0–52 [42]), kinesiophobia (TSK), self-efficacy despite pain (Self-Efficacy Scale, SES, 0–200, [43]), the Work Ability Index (WAI, 7–49 [26]), anxiety and depression (HADS), and general pain-related disability [Pain Disability Index (PDI (0–70)] [44]. For the analysis of factors associated with change in the EQ score, the change scores of these measurements were used, and randomization group and adherence (more or less than 50%) were also added.

### Statistics

The sample size was based on the primary outcome of the main study (the NDI) as previously reported [22]. Descriptive statistics and group comparisons with one-way ANOVA, the Kruskal–Wallis test or *Χ*^2^ test as appropriate were used for baseline characteristics. The EQ-5D can be regarded as a continuous outcome [45]. Linear mixed models were used to analyse the EQ-5D score/VAS and both SF-36 summary scores, including all available data at all four time points, and three group levels. A factor analytic, first-order heterogeneous covariance matrix was used, with randomization group and time as fixed factors. To control for the significant differences between groups, gender and age were added as covariates. The Bonferroni correction was used for post hoc estimated marginal means main effects.

To test factors associated with change in the HRQoL scores, bivariate correlations were first tested with Spearman’s (non-parametric) test and Pearson’s (parametric and biserial for gender) test. The purpose of these analyses was to find out which factors to include in the multivariate linear regression model. Significant variables were entered into a multivariate linear regression model after checking independent variables for collinearity with linear regressions for each variable. There was no collinearity between any of the independent variables (all variance inflation factors < 5 and tolerance levels > 0.36). The multivariate linear regression of significant factors was performed with stepwise backward regression, with *p* ≥ 0.1 as a limit for removal of variables to reduce the risk of overlooking potential important factors.

The significance level was set at *p* ≤ 0.05. Analyses were performed with SPSS 23.0 (SPSS Inc., Chicago, IL, USA).

## Results

### Adherence and drop-out analysis

The percentage of adherent participants (at least 50% attendance) during the 12-week intervention period was 70% and 71% in the NSE and NSEB groups and 47% in the PPA group (*p* = 0.07). The questionnaires were completed by 170–185 participants (79–85%) at the follow-ups (Fig. 1). There was no difference between completers and 12-month dropouts regarding baseline EQ-5D score, EQ-VAS, PCS/MCS, gender, age or comorbidities (all *p* > 0.29). No serious adverse events were reported but two participants dropped out due to increased pain (Fig. 1), which may be associated with registered deviation from the exercise protocol.

### Results of the randomized study

Mean scores and *p* values of differences between groups and over time are presented in Table 2.

**Table 2:** Health-related quality of life scores from baseline to 12 months

	*n*	Mean EQ-5D score								
		Baseline	95% CI	3 months	95% CI	6 months	95% CI	12 months	95% CI	*p* value
NSE	76	0.649 (0.217)	0.599–0.698	0.685 (0.233)	0.626–0.745	0.680 (0.258)	0.611–0.749	0.708 (0.243)	0.643–0.773	0.52
NSEB	70	0.552 (0.307)	0.478–0.625	0.686 (0.212)	0.633–0.738	0.704 (0.250)	0.638–0.771	0.673 (0.276)	0.602–0.745	< 0.01
PPA	69	0.631 (0.249)	0.570–0.692	0.622 (0.291)	0.545–0.699	0.627 (0.285)	0.547–0.707	0.618 (0.289)	0.536–0.639	0.90

	*n*	Mean EQ-VAS								
		Baseline	95% CI	3 months***	95% CI	6 months	95% CI	12 months***	95% CI	*p* value
NSE	75	65 (16)	60–69	70 (18)	63–74	70 (21)	65–76	70 (21)	64–76	0.14
NSEB	70	61 (20)	54–65	66 (20)	61–73	71 (20)	66–78	71 (20)	67–78	< 0.01
PPA	69	64 (18)	60–69	61 (21)	54–66	64 (20)	59–70	62 (21)	55–67	0.12

	*n*	Mean SF-36 physical component score								
		Baseline	95% CI	3 months***	95% CI	6 months	95% CI	12 months***	95% CI	*p* value
NSE	74	42.54 (6.41)	40.66–44.41	45.44 (7.40)	43.24–47.63	45.93 (7.93)	43.24–47.23	46.01 (8.46)	43.65–48.54	< 0.01
NSEB	70	43.02 (6.80)	41.15–44.48	44.85 (6.76)	42.98–46.91	45.46 (7.25)	43.49–47.42	46.22 (7.56)	44.15–48.28	< 0.01
PPA	69	42.14 (6.23)	40.26–44.01	42.95 (9.20)	40.23–45.68	43.89 (7.70)	41.63–46.14	42.72 (8.47)	40.21–45.23	0.33

	*n*	Mean SF-36 mental component score								
		Baseline	95% CI	3 months***	95% CI	6 months	95% CI	12 months	95% CI	*p* value
NSE	74	47.89 (8.74)	44.43–51.34	49.37 (12.74)	44.35–54.38	49.75 (9.63)	45.98–53.53	50.71 (10.82)	46.56–54.87	0.38
NSEB	70	47.17 (10.73)	43.97–50.37	49.20 (10.05)	46.14–52.26	49.48 (11.23)	46.16–52.80	48.71 (13.44)	44.54–52.89	0.21
PPA	65	46.50 (12.03)	41.83–51.17	47.37 (11.42)	43.02–51.73	46.09 (13.57)	40.85–51.32	46.21 (12.74)	41.39–51.04	0.91

Adjusted for age and gender

*NSE* Neck-specific exercise group, *NSEB* Neck-specific exercise group with a behavioural approach, *PPA* physical activity group, *EQ-5D* Euroqol 5 dimension questionnaire, *SF-36* Short Form health questionnaire 36, *CI* confidence interval

***p < 0.01 difference between groups

Regarding the EQ-5D score, there was a significant group-by-time interaction (*F* = 3.1, *p* < 0.01) with significant difference over time (*F* = 3.3, *p* = 0.02). There was a significant improvement in the NSEB group from baseline to three months, which was maintained over all time points (*F* = 5.1, *p* < 0.01). However, the changes in the NSE (*F* = 1.6, *p* = 0.20) and PPA groups (*F* = 0.1, *p* = 0.9) were insignificant. There was no difference between groups (*F* = 1.3, *p* = 0.28) (Table 2).

Regarding the EQ-VAS, there was a significant group-by-time interaction (*F* = 4.3, *p* = 0.49) with significant difference over time (*F* = 4.3, *p* < 0.01) and between groups (*F* = 10.0, *p* < 0.01). There were significant differences between both the NSE and NSEB groups compared to the PPA group (NSE, NSEB *p* < 0.01) at 3 and 12 months. The NSEB group improved over time (*F* = 8.0, *p* < 0.01), and the significant change at 6 months was maintained at 12 months. The changes over time in the NSE (*F* = 1.9, *p* = 0.14) and PPA (*F* = 2.0, *p* = 0.12) groups were insignificant (Table 2).

Regarding the SF-36 PCS, there was a significant difference between groups (*F* = 12.7, *p* < 0.01) and time (*F* = 4.7 *p* < 0.01). There were significant differences between both the NSE and NSEB groups compared to the PPA group (*p* < 0.01). Both the NSE/NSEB groups improved over time (NSE *F* = 6.5, *p* < 0.01, NSEB *F* = 6.6, *p* < 0.01), as opposed to the PPA group (*p* = 0.33). The NSE group remained improved at all follow-ups, and the NSEB group had improved at the 6- and 12-month follow-ups. The group-by-time interaction effect was insignificant (*F* = 0.9, *p* = 0.48) (Table 2).

For the MCS, there was a significant group difference (*F* = 5.9, *p* < 0.01), between both the NSE and NSEB groups compared to the PPA group (NSE *p* = 0.03, NSEB *p* < 0.01) at 3 months. The time (*F* = 0.4, *p* = 0.78) and interaction effects (*F* = 0.7, *p* = 0.65) were insignificant (Table 2).

There were no differences between the NSE/NSEB groups in any of the HRQoL outcomes at any time point.

At baseline, 69% of the participants reported comorbidity (*n* = 148). The two most common comorbidities were psychological distress (24%, *n* = 51) and pulmonary disease, e.g. asthma/bronchitis (16%, *n* = 34). Participants who reported comorbidities tended to report worse health than those without: EQ-5D score 0.593 (SD 0.270) versus 0.653 (SD 0.240), *p* = 0.12, EQ-VAS 62 (SD 17) versus 67 (SD 18) mm, *p* = 0.05, PCS 42.3 versus 44.1 and MCS 45.2 versus 49.9, *p* = 0.07 for both.

### Factors associated with the HRQoL scores and change scores

#### Baseline score

At baseline, a bivariate significant correlation was found between the different HRQoL measurements and most scores and comorbidity. Months since injury was not associated with any of the outcomes, whereas for instance age, educational level, pain, depression and WAD grade were correlated with some of the HRQoL outcomes (Supplement 1).

The significant factors (presented in Supplement 1) were used in the multivariate linear models, and the results of the final models explaining 43–59% of the variance are presented in Table 3. When considering the influence of all other factors, HADS depression was the only factor significantly associated with all four HRQoL outcomes. It was negatively associated, i.e. higher levels of depression predict lower HRQoL with an EQ-5D score of 0.02 or 1.27 mm on the EQ-VAS per HADS point. Work ability (WAI) and pain disability (PDI) were associated with three out of four outcomes.

**Table 3:** Factors associated with HRQL baseline and 6-month change scores, results of final multivariate models

Factors associated with	Baseline EQ-5D score (*R*^2^ = 0.52)			Baseline EQ-VAS (*R*^2^ = 0.43)			Baseline SF-36 PCS (*R*^2^ = 0.59)			Baseline SF-36 MCS (*R*^2^ = 0.45)		
	*b* (SE)	*β*	*p*	*b* (SE)	*β*	*p*	*b* (SE)	*β*	*p*	*b* (SE)	*β*	*p*
WAI	0.01 (0.00)	0.34	< 0.01	0.80 (0.22)	0.30	< 0.01	0.27 (0.08)	0.28	< 0.01	n.a	n.a	n.a
HADS depression	− 0.02 (0.01)	− 0.325	< 0.01	− 1.27 (0.33)	− 0.29	< 0.01	− 0.42 (0.11)	0.27	< 0.01	− 1.14 (0.26)	− 0.24	< 0.01
HADS anxiety	n.a	n.a	n.a	n.a	n.a	n.a	n.a	n.a	n.a	− 0.50 (0.21)	− 0.19	0.02
Comorbidities, numbers	0.03 (0.01)	0.125	0.03	n.a	n.a	n.a	n.a	n.a	n.a	n.a	n.a	n.a
PDI	− 0.004 (0.02)	− 0.228	0.01	n.a	n.a	n.a	− 0.87 (0.43)	− 0.18	0.04	− 0.18 (0.80)	− 0.21	0.03
PCSc	− 0.004 (0.02)	− 0.174	0.01	n.a	n.a	n.a	n.a	n.a	n.a	n.a	n.a	n.a
SES	− 0.001 (0.001)	− 0.195	0.02	n.a	n.a	n.a	0.02 (0.01)	0.13	0.09	n.a	n.a	n.a
Age	n.a	n.a	n.a	n.a	n.a	n.a	− 0.71 (0.31)	− 121	0.02	n.a	n.a	n.a
NDI	n.a	n.a	n.a	n.a	n.a	n.a	− 0.42 (0.9)	− 0.44	< 0.01	0.44 (0.16)	0.27	0.01
Pain VAS	n.a	n.a	n.a	− 0.16 (0.53)	− 0.21	< 0.01	n.a	n.a	n.a	n.a	n.a	n.a

Factors associated with	EQ-5D change score (R^2^ = 0.42)			EQ-VAS change (*R*^2^ = 0.41)			SF-36 PCS change (R^2^ = 0.45)			SF-36 MCS change (R^2^ = 0.38)		
	*b* (SE)	*β*	*p*	*b* (SE)	*β*	*p*	*b* (SE)	*β*	*p*	*b* (SE)	*β*	*p*
Educational level	0.07 (0.03)	0.19	0.01	n.a	n.a	n.a	n.a	n.a	n.a	n.a	n.a	n.a
HADS depression change	0.03 (0.01)	0.36	< 0.01	3.00 (0.57)	0.44	< 0.01	n.a	n.a	n.a	0.86 (0.25)	0.32	0.01
PCSc change	0.01 (0.0)	0.22	0.02	n.a	n.a	n.a	n.a	n.a	n.a	n.a	n.a	n.a
VAS change	0.002 (0.0)	0.18	0.03	n.a	n.a	n.a	n.a	n.a	n.a	n.a	n.a	n.a
WAI change	n.a	n.a	n.a	1.01 (0.58)	0.30	0.01	0.18 (0.09)	0.18	0.057	0.33 (0.14)	0.25	0.02
NDI change	n.a	n.a	n.a	n.a	n.a	n.a	0.28 (0.05)	0.54	< 0.01	0.12 (0.07)	0.17	0.01

Results of the final regression models

*EQ-5D* Euroqol 5 dimensions health questionnaire, *SF-36* Short Form 36 health questionnaire, *PCS* Physical component summary, *MCS* mental component summary, *HADS* Hospital Anxiety and Depression Scale, *PDI* Pain disability score, *PCSc* Pain Catastrophizing Scale, *SES* Self-Efficacy Scale, *VAS* Visual Analogue Scale, *WAI* Work Ability Index, *NDI* Neck Disability Index, *n.a*. not applicable due to variable not included in the final step of the regression. *β* standardized regression coefficient, *b* unstandardized coefficient, *SE s*tandard error

#### Change scores from baseline to 6 months

For change scores from baseline to 6 months, univariate significant correlations were found between the different HRQoL change scores and the change scores of most outcomes. No correlation was found between the HRQoL change scores and educational level, months since injury or adherence. Age, WAD grade and randomization group were correlated with some of the outcomes, and gender and comorbidity were close to a significant correlation with the EQ-5D index (*p* = 0.053–0.06) (Supplement 1).

The significant factors were used in the multivariate linear models, and the results of the final models, explaining 38–45% of the variance, are presented in Table 3. No factors were associated with all four HRQoL outcomes, but reduction of depression was associated with improvements in all but the PCS. A decrease of the level of depression was associated with an increase of HRQoL with an EQ-5D score of 0.03 or 3 mm on the EQ-VAS per HADS point. Apart from depression, improvement of both the physical and mental aspects of SF-36 was the same (improvement of work ability (WAI) and neck disability (NDI)). Improvement of work ability was also associated with improvement of EQ-VAS, but it was not associated with the EQ-5D index, which instead was associated with educational level, pain and pain catastrophizing.

## Discussion

The results indicate that NSE/NSEB may improve HRQoL compared to PPA in chronic WAD grade 2 or 3, especially when combined with a behavioural approach. There was no difference between groups regarding the EQ-5D score, although the NSEB group improved over time. This was also the case with EQ-VAS, but there was also a group difference favouring the NSE/NSEB groups compared to PPA. Regarding the SF-36 PCS, both the NSE and NSEB groups improved over time. There was also a group difference favouring these two groups over PPA, which was also the case for the SF-36 MCS at 3 months, but the MCS improvement over time was insignificant. Although there were no differences between the NSE/NSEB groups, there was a tendency towards larger improvements with the addition of a behavioural approach to the NSE, which—given the overlap between psychosocial and physical symptoms—could be expected. There were no improvements in HRQoL in the PPA group.

None of the group means reached the norm HRQoL levels of the general public. However, the improvements of the SF-36 PCS in the NSE/NSEB groups (3.47/3.2) were above the suggested minimal clinical important difference (MCID) (2.7) after non-surgical treatment in chronic neck pain [46]. This suggests that the improvements were nonetheless clinically relevant. Regarding EQ-5D we have not been able to find a suggested level of MCID in chronic neck pain following non-surgical interventions. In chronic pain including rheumatoid arthritis, post-herpetic neuralgia, lumbago, etc., the overall MCID of EQ-5D is suggested to be 0.1 following introduction of pain medication [47]. Since the MCID depends on the kind of disease/pain condition and of the intervention in question, this level may not be true in chronic WAD.

NSE/NSEB seem to significantly reduce a number of neck-related problems in longstanding WAD grades 2 and 3 [22, 48–51], but the perception of health is complex. For a person with chronic WAD, not only neck symptoms but also many other factors are associated with HRQoL, as shown in Table 3, and Supplement 1. More than two-thirds of the participants in this study reported comorbidity, which was correlated with HRQoL just like most measurements in the univariate analyses. However, the results of the multivariate analyses indicate that when considering the influence of all other factors, the only factor associated with all four baseline outcomes was depression. The significance of depression is in line with previous literature [11, 12]. Work ability and pain disability were also associated with three out of four HRQoL baseline outcomes.

Baseline factors may predict an outcome, but they do not explain whether these factors may also mediate the outcome. To our knowledge, factors associated with *change* in HRQoL (i.e. factors that can explain the improvement) following exercise interventions in chronic WAD grades 2 and 3 have not been presented before. As seen in Table 3, the factors associated with HRQoL and change of HRQoL are not identical. For instance, age only explained part of the variance in the baseline PCS score, whereas it did not seem to play a role in the improvement of health. On the other hand, factors like depression and work ability were associated with both HRQoL and change of HRQoL. Reducing the level of depression thus seems to be an important factor to consider. Exercise is reported to be more effective at reducing depression in people classified as depressed than those within the normal range [52]. Even though the mean baseline level of depression in this sample indicated no depression (Table 1), 67 participants reported at least borderline scores (HADS depression > 7). When separating the physical aspect from the mental aspect of HRQoL, improvement of depression was only associated with improvement of the mental aspect. Improvements of both work ability and neck disability were however associated with improvements of both the physical and mental aspects according to the SF-36. Factors associated with improvements of the EQ-5D score were partly different, where educational level, improvements of both pain and pain catastrophizing seem to play a larger role than work ability and neck disability. The NSE and/or the NSEB groups have gained larger improvements in pain catastrophizing [48], neck disability, pain [22] and work ability [53] than the PPA group as previously presented in this sample, which may be part of the explanation of the better results of the NSE/NSEB groups also regarding HRQoL. Taking the multidimensional nature of health into account, the *R*^2^values, explaining the variance of the outcome, were relatively high. It should be noted that no single factor alone can explain the whole variance. The *R*^2^ values are based on a combination of several factors associated with HRQoL, as seen in Table 3, and relate to the included variables only. Other factors, not included in this analysis may also play an important role.

Generic measurements, like the EQ-5D, are not only used to report HRQoL, but are also used in cost-effectiveness analyses. Generic measurements are however generally less responsive than disease-specific measurements [54]. Although improvements were seen in the NSE/NSEB groups in this study and NSE seems to be cost-effective compared to NSEB and PPA in chronic WAD [30], caution is warranted when comparing with other groups of patients. Both the SF-36 and EQ-5D have a greater focus on lower body function than on upper body function, which may reduce the sensitivity in WAD populations and have an impact when comparing different health states. Furthermore, the EQ-5D score, with its fewer items, is less sensitive than the SF-36 [55], but the EQ-VAS also offers an option to consider other aspects not covered by the questions.

## Limitations

Since this is a secondary analysis, the sample-size calculation was not based on HRQoL, and there may thus have been insufficient power to detect more differences. There was a small, but significant, baseline difference between groups regarding age and gender; however, they were not correlated with the change scores. Furthermore, they were controlled for in the analysis.

Another limitation is that even though the physiotherapists were experienced and selected to match their interest and competence as far as possible, the one-day training may not have been enough to fully master the interventions. Nonetheless, an important psychological factor, pain catastrophizing, was, as previously presented, improved by up to a mean of 37% following NSE alone and 30% following NSEB [48].

Finally, participants in the PPA group only had 1–2 physiotherapist visits, whereas the other two groups had regular physiotherapist contact which may have influenced the results. However, expectations of the three interventions were similar at baseline and there was no difference between groups regarding fulfilment of expectations [22].

## Conclusions

Neck-specific exercise, particularly with a behavioural approach, may have a more positive impact on HRQoL than physical activity prescription in chronic WAD grades 2 and 3. HRQoL is however complex, and other factors also need to be considered. The factors associated with HRQoL and improvements in HRQoL (*R*^2^ = 0.38–0.50) following exercise were multidimensional.

## Electronic supplementary material

Below is the link to the electronic supplementary material.

Supplementary material 1 (DOCX 15 KB)
