# Supplementary material for: The effect of three exercise approaches on health-related quality of life, and factors associated with its improvement in chronic whiplash-associated disorders: analysis of a randomized controlled trial
Source: Qual Life Res. 2018 Sep 17;28(2):357–68. doi: 10.1007/s11136-018-2004-3 (PMC6373318; doi:10.1007/s11136-018-2004-3)
Supplement: Supplementary file 1 — Supplementary material 1 (DOCX 15 KB) [file 11136_2018_2004_MOESM1_ESM.docx]

**Supplement 1.** Factors with bivariate significant correlation with HRQL measurements, included in the multivariate regression analysis.

|  | **Factors correlated with baseline scores** | | | |
| --- | --- | --- | --- | --- |
| **C*orrelating baseline factors*** | **EQ-5D score** | **EQ VAS** | **SF-36 PCS** | **SF-36 MCS** |
| Age | - | - | x | - |
| Gender | - | - | - | - |
| Months since injury | - | - | - | - |
| Educational level | x | - | - | - |
| WAD grade | x | x | x | - |
| Comorbidity | x | x | x | x |
| Neck pain VAS | x | x | - | - |
| NDI | x | x | x | x |
| PDI | x | x | x | x |
| TSK | x | x | x | x |
| HADS depression | x | x | - | x |
| HADS anxiety | x | x | x | x |
| PCSc | x | x | x | x |
| SES | x | x | x | x |
| WAI | x | x | x | x |
|  |  |  |  |  |
|  |  |  |  |  |
|  | **Factors correlated with change scores** | | | |
| ***Correlating baseline factors*** | **EQ-5D score** | **EQ VAS** | **SF-36 PCS** | **SF-36 MCS** |
| Age | (x) | - | x | x |
| Gender | - | - | - | - |
| Months since injury | - | - | - | - |
| Educational level | - | - | - | - |
| WAD grade | - | x | - | - |
| Comorbidity | (x) | - | - | - |
| Randomization group | - | x | - | - |
| Adherence | - | - | - | - |
| ***Correlating change scores:*** |  |  |  |  |
| Neck pain VAS | x | x | x | x |
| NDI | x | x | x | x |
| PDI | x | x | x | x |
| TSK | x | x | x | x |
| HADS depression | x | x | x | x |
| HADS anxiety | x | x | x | x |
| PCSc | x | x | x | x |
| SES | x | x | x | - |
| WAI | x | x | x | x |

Significant (p<0.05) bivariate correlations with Health related quality of life outcomes. EQ-5D = Euroqol 5 Dimensions health questionnaire, SF-36 = Short Form 36 health questionnaire, PCS = Physical Component Summary, MCS = Mental Component summary, HADS = Hospital Anxiety and Depression scale, PDI = Pain Disability Score, PCSc = Pain Catastrophizing scale, SES = Self-Efficacy Scale, VAS= Visual Analogue Scale, WAI= Work Ability Index, NDI = Neck Disability Index, (x) = included in the multivariate analysis, but bivariate p= 0.06-0.07
